# What was the primary mode of smallpox transmission? Implications for biodefense

**DOI:** 10.3389/fcimb.2012.00150

**Published:** 2012-11-29

**Authors:** Donald K. Milton

**Affiliations:** ^1^Maryland Institute for Applied Environmental Health, School of Public Health, University of MarylandCollege Park, MD, USA; ^2^Department of Medicine, University of Maryland School of MedicineBaltimore, MD, USA; ^3^Department of Environmental Health, Harvard School of Public HealthBoston, MA, USA

**Keywords:** smallpox, bioterrorism, biodefense, variola virus, air microbiology, communicable diseases, airborne infection transmission, contact infection transmission

## Abstract

The mode of infection transmission has profound implications for effective containment by public health interventions. The mode of smallpox transmission was never conclusively established. Although, “respiratory droplet” transmission was generally regarded as the primary mode of transmission, the relative importance of large ballistic droplets and fine particle aerosols that remain suspended in air for more than a few seconds was never resolved. This review examines evidence from the history of variolation, data on mucosal infection collected in the last decades of smallpox transmission, aerosol measurements, animal models, reports of smallpox lung among healthcare workers, and the epidemiology of smallpox regarding the potential importance of fine particle aerosol mediated transmission. I introduce briefly the term anisotropic infection to describe the behavior of Variola major in which route of infection appears to have altered the severity of disease.

## Introduction

Controversy exists regarding the best method of protecting the public against the potential release of smallpox as a biological weapon (Bicknell, 2002; Fauci, 2002; Halloran et al., 2002; Kaplan et al., 2002; Mack, 2003). Infectious disease modeling plays an important role in this dialog, and the biology of the transmission pathway, the focus of this review, is critical to producing appropriate predictive models and understanding which controls will work best under varying conditions (Ferguson et al., 2003).

The rapidity with which smallpox would spread in a developed nation is not known and is a major source of uncertainty in models used for public health planning (Ferguson et al., 2003). The basic reproductive number (R_0_), which describes the tendency of a disease to spread, has been estimated for smallpox from historical data and outbreaks in developing countries (Gani and Leach, 2001; Eichner and Dietz, 2003). Because R_0_ is a function of the contact rate between individuals, it can be affected by changes in the environment (Anderson and May, 1991). A potentially important difference between contemporary environments and those used to estimate R_0_ is that today many buildings, including hospitals, mechanically recirculate air. If smallpox was almost entirely transmitted by mucosal contact with large droplets (aerodynamic diameters >10 μm), which can only occur following “face-to-face” exposure over distances of a few feet, then change in the built environment would not change the contact rate between individuals. If, however, smallpox was frequently transmitted from person-to-person by airborne droplet nuclei [fine particles with aerodynamic diameters of ≤2.5 μm capable of remaining suspended in air for hours and of depositing in the lower lung (Hinds, 1999)] then mechanically recirculated air systems would increase the contact rate, R_0_, the risk of epidemic spread, and the difficulty of hospital infection control. Unfortunately, leading authorities disagree regarding the relative importance of fine and large particle routes of transmission; some state that smallpox was transmitted primarily via airborne droplet nuclei, (Henderson et al., 1999) while others emphasize “face-to-face” contact and state that, airborne transmission was rare (Centers for Disease Control, 2002; Mack, 2003). This paper reviews the evidence for each of these modes of transmission.

## Variolation

Prior to Jenner, variolation, (Fenner et al., 1988) inoculation of variola into the skin or nasal mucosa, was used to reduce the risk of smallpox. Jenner himself was variolated as a child. Skin inoculation with a small amount of fresh pustule fluid, likely to have contained large numbers of infectious virions, produced a local lesion with satellite pustules, but generalized rash was reported to be less severe and mortality rates were usually 10-fold lower than with naturally acquired disease (Fenner et al., 1988). In China, variolation was frequently performed by inoculation of the nasal mucosa. Some accounts describe blowing carefully aged scabs compounded with plant material into the nose (MacGowan, 1884). Other reports suggests that nasal insufflation was considered relatively ineffective and that nasal insertion of cotton pledgets impregnated with powdered scabs or smeared with vesicle contents was preferred (Wong and WU, 1936; Miller, 1957). Descriptions of the latter method do not include ageing infectious material before use.

**Figure 1:**
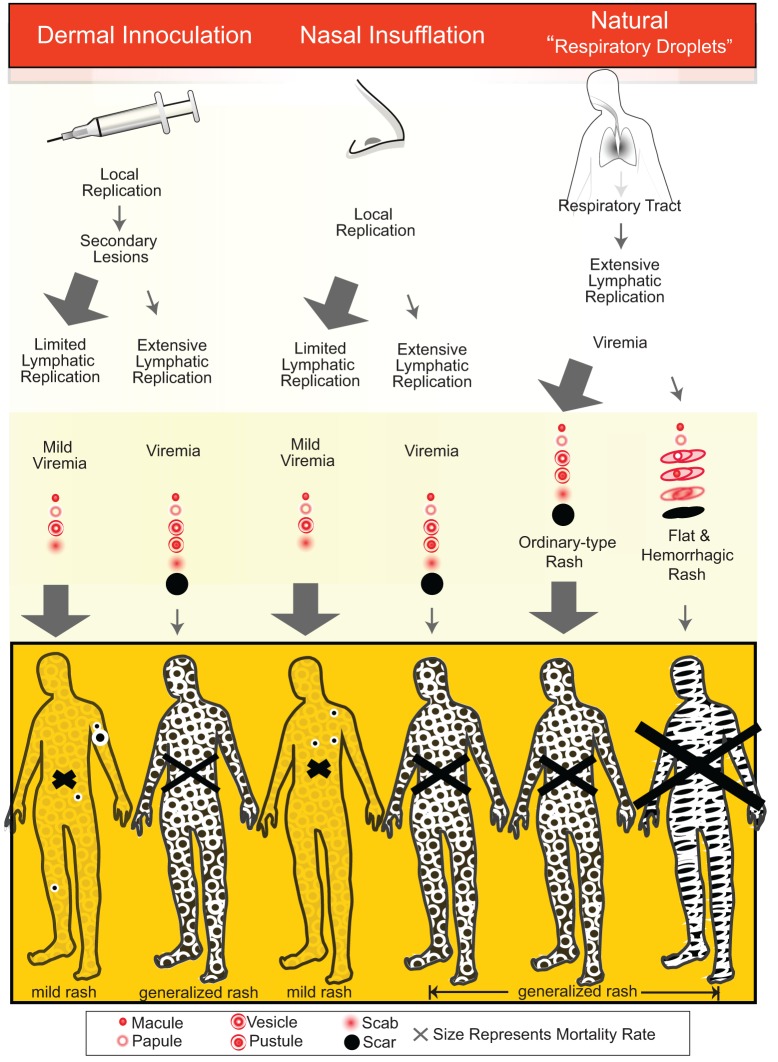
**The spread of variola virus around the body [partially adapted from Fenner et al. (1988) Figure 3.1] appears to have frequently been less extensive after dermal inoculation and nasal insufflation compared with naturally acquired infection.** This may have been due to less extensive lymphatic replication of virus and limited viremia by dermal and nasal routes as compared with infection via lower respiratory tract deposition. The size of the arrows represents the historically reported proportions of cases following each pathway. The size of the X on each image represents the reported mortality rate from each pathway. For natural infection, the ordinary-type rash and flat and hemorrhagic rashes are shown.

Because natural infection was thought to occur via large droplets deposited on the upper respiratory mucosal, the success of nasal inoculation in producing low mortality rates has been hard to understand. A theory suggested by Henderson to the author of a smallpox history, (Hopkins, 1983, p. 114) “is that virus inhaled naturally was in sufficiently small particles to be deposited deep within the lung, whereas particles inoculated by nasal insufflation may have been much larger and were likely to implant in the nose or throat where [only] a local lesion might be produced.” The relative importance of age and health of inoculated subjects, infectious dose, and route of exposure are not known. However, it appears that inoculation via the skin or nasal mucosa tended to produce modified disease. If true, this would indicate that natural transmission did not occur via direct skin or mucosal contact. Figure 1 shows graphically a how these different routes of exposure may have produced altered patterns of viral replication within the host and resulted in different risks of extensive viremia and severe disease.

## The paradox of mucosal infection

If natural smallpox was initiated through the upper respiratory mucosa, then an early asymptomatic mucosal infection would be expected. To investigate this, Sarkar and colleagues performed pharyngeal swab surveys of household contacts (Sarkar et al., 1973a, 1974) 4–8 days following onset of rash in the index cases. They found that contacts with positive throat cultures often did not develop smallpox. In one survey, (Sarkar et al., 1973a) 10% (Westwood et al., 1966) of 328 contacts had positive swabs, but only 12% (Kaplan et al., 2002) of those with positive swabs developed smallpox. Among 59 unvaccinated contacts 27% (Miller, 1957) were culture positive, but only one developed smallpox. All subjects were vaccinated at the time of examination. However, vaccination four or more days after exposure is usually considered to be too late to prevent disease. The observation that disease did not develop in 94% of persons with mucosal infection suggests that, even in unvaccinated contacts, mucosal infection may not have been sufficient to initiate disease.

Sarkar and colleagues also showed that the oropharyngeal excretion of virus was greatest during the first days after the rash erupted and generally resolved at most 2 weeks following onset of rash (Sarkar et al., 1973b). Rao et al. found that oropharyngeal excretion was greatest in the most severe, hemorrhagic cases and corresponded with the period of infectiousness (Rao et al., 1968). In contrast to oropharyngeal excretion, scabs contained large quantities of virus regardless of disease severity (Mitra et al., 1974) and were shed for another week or more after throat cultures were negative. Scabs alone, however, were not associated with further cases (Rao et al., 1968; Mitra et al., 1974).

The apparent lack of infectiousness of scab associated virus has been attributed to encapsulation with inspissated pus (Fenner et al., 1988). Henderson's theory about the importance of small particles may provide a straightforward mechanism for why encapsulated virus, simply by entrapment in large particles, had low infectious potential.

Sarkar et al. (1973a) were concerned that asymptomatic contacts could have been infectious because their throat swab viral titers were similar to those of milder smallpox cases. A paradox arose from these data because there was never evidence of infection arising from asymptomatic household contacts. Yet, oropharyngeal secretions were thought to be the primary source of infectious virus particles. An explanation may be that oropharyngeal excretion of virus was merely temporally correlated with excretion of virus from elsewhere in the respiratory tract and not the actual source of fine particles virus aerosols.

The large spray of particles from sneezing visualized by high speed photography consists of particles down to about 10 μm in diameter (Papineni and Rosenthal, 1997). Smaller particles may also be dislodged from the upper airways by the turbulence of sneezing, coughing, and talking, but will mostly be larger than 2.5 μm in diameter. Recent studies, however, show that the healthy lung generates abundant fine particles (100–1000/l with size <0.3 μm diameter) during normal breathing (Fairchild and Stampfer, 1987) that do not arise from the oropharynx; condensates of these particles are the subject of recent reviews (Mutlu et al., 2001; Hunt, 2002). Such particles could carry variola virus (0.2–0.3 μm diameter), would remain airborne in indoor air for many hours, and would be deposited primarily in the lower airways after inhalation.

There is some evidence that variola was present in the lung and potentially available for aerosolization. Animals infected by inhalation produced high concentrations of variola in the lung (Hahon and Wilson, 1960). Fenner et al. (1988) regarded bronchitis and pneumonitis as a part of the normal smallpox syndrome, especially in the more severe cases which were also the most infectious, (Rao et al., 1968) although specific lesions were less frequent in the lower trachea and bronchi. Systematic evaluations of viral excretion in the lower respiratory tract of non-fatal cases were not reported. Thus, if some degree of pneumonitis with pulmonary excretion of virus and exhalation of fine particle variola aerosols was a feature of clinical smallpox but was not a feature asymptomatic household contact with positive throat cultures, then the paradox would be resolved.

## Measurement and half-life of airborne variola

Air sampling for viruses is a difficult undertaking and the literature on the subject remains sparse in comparison with that for bacteria and fungi (Sattar and Ijaz, 2002). Only three attempts to detect airborne variola were published. The earliest attempt used highly inefficient methods and was negative (Meiklejohn et al., 1961). In a subsequent study, Downie and colleagues used short duration, low volume air sampling with liquid impingers and obtained 5 positive samples out of 47 attempts to sample exhaled breath of patients (Downie et al., 1965). Assuming that each positive sample represented a single infectious particle, the concentration of airborne infectious particles was 0.85/m^3^; higher concentrations were observed close to shaken bed sheets. Concentrations were likely to have been underestimated because of several frequently encountered problems with air sampling for viruses including failure of impingers to retain particles less than 1 μm in diameter that represent the majority of particles in exhaled breath, culture of only a portion of the impinger fluid, uncertain suitability of sampling fluid for virus survival, and loss of infectivity due to sampling trauma (Spendlove and Fannin, 1982).

In the 1970s, Thomas adapted Andersen samplers (capable of colleting submicrometer particles) and slit samplers (with lower efficiency for submicrometer particles) for long duration large air volume viral sampling (Thomas, 1970a). He showed that 23% of naturally airborne rabbit pox particles were ≤2.5 μm and 71% were between 2.5 and 10 μm (Thomas, 1970b). Both Thomas and Westwood et al. (1966) measured concentrations of natural rabbit pox aerosols. Thomas observed 12 pock forming units (PFU) per m^3^ in a room supplied with six air changes per hour (ACH) containing 27 ill rabbits. Westwood et al. observed 44 PFU/m^3^ in a room supplied with 10 ACH containing 7–9 infected rabbits. Westwood et al. probably obtained higher concentrations because they used an electrostatic precipitator allowing higher efficiency collection of submicrometer particles compared with Thomas's slit sampler.

Thomas also studied convalescent cases of variola minor (Thomas, 1974). One patient with relatively active lesions produced an average concentration of approximately 1 PFU/m^3^. Unfortunately the samples were collected late in the disease when the patient was probably minimally infectious, based on comparison with epidemiological data (Rao et al., 1968; Eichner and Dietz, 2003). The airborne virus observed appears to have been due to resuspension and is unlikely to be representative of the airborne concentration of respirable variola earlier in the course of the infection. The method used would also not have been able to collect submicrometer viral aerosol particles.

Overall, the air sampling studies suggest that animals and people infected with poxviruses generated respirable aerosols, but that air concentrations may have been low, or airborne virus was present in submicrometer particles that could not be collected the instruments available. Because detection of virus aerosols is subject to potentially large losses in sampling equipment, especially when sampling dilute natural aerosols over extended periods, and because plaque assays may not accurately represent the infectivity of virus deposited in human airways at 100% relative humidity, (Spendlove and Fannin, 1982; Sattar and Ijaz, 1987, 2002) the available data can be considered a lower limit on concentration of infectious natural poxvirus aerosols.

Experimental aerosol data suggested that poxvirus, which survived the trauma of artificial aerosolization, remained infectious for significant periods of time. Aerosols of vaccinia demonstrated a half-life of about 6 h at 22°C and relative humidity ≤50% with reduced stability at higher relative humidity and temperature (Harper, 1961). Variola appeared to have a similar half-life and not to be affected by relative humidity at 26.67°C (Mayhew and Hahon, 1970). Other experiments demonstrated that airborne vaccinia is highly sensitive to inactivation by germicidal ultraviolet light (Edward et al., 1943; Jensen, 1964).

## Animal models

Westwood et al. (1966) demonstrated that inhalation of a single PFU of a submicrometer vaccinia aerosol was sufficient to infect rabbits. Airborne rabbit pox was similarly infectious. They demonstrated rabbit-to-rabbit airborne transmission of rabbit pox in each of seven trials by placing uninfected rabbits in separate cages in the same room with infected animals. They also infected rhesus monkeys using submicrometer aerosols of variola.

In one of the earliest extensive animal models of smallpox, Brinckerhoff and Tyzzer (1906) reported the effect of inoculating cynomologus monkeys with variola at different sites. Inoculation of mucus membranes of the lip, palate, and nose produced local lesions, but generalized rash occurred in only 10% of animals. Inoculation through the skin produced a local lesion and a generalized eruption in 70–80% of animals. Animals inoculated by scratching the tracheal mucosa through a rigid bronchoscope all developed a generalized rash, and one developed a variolous bronchitis and pneumonia. Laryngeal instillation of dry pustule contents produced infections while instillation of powdered crusts did not. Inhalation exposures to an atomizer spray of vesicle contents infected only one of five monkeys; however, the particle size distribution and type of atomizer were not reported.

Hahon and Wilson demonstrated that infection of *Macaca irus* with high dose [5 × 10^5^ PFU] fine particle (<5 μm) variola aerosols produced a disease that simulated human smallpox (Hahon and Wilson, 1960; Hahon, 1961). The initial site of virus replication was the lung, with subsequent appearance of virus in the nasopharynx and nares. Peak concentrations of virus per gram of tissue were higher in the lung than in the upper respiratory tract; the peak in lung tissue occurred during the incubation period and lung levels declined during the secondary viremia and exanthem. Whether the time course and viral concentrations in lung in this animal model produced by inhalation of high dose aerosols mimicked that in humans with natural infection is doubtful. However, it may be relevant to the first generation of cases exposed to concentrated aerosols in a biological attack. In a relatively recent experiment, (Kalter et al., 1979) a female chimpanzee became infected with variola while housed in the same room, but without direct contact, with two infected chimpanzees. She developed a generalized rash and was reported to have had more severe constitutional symptoms than the other chimpanzees infected by dermal inoculation or direct contact. The authors concluded that she was infected via aerosol.

The animal data show that artificial respirable aerosols were effective means of producing poxvirus infections, that the infectious dose by the airborne route could be very low, and that animal-to-animal airborne transmission of rabbitpox and variola was observed. They also suggest that inoculation of mucus membranes was less effective at producing a generalized rash than was exposure of the lower respiratory tract.

## “Smallpox handler's lung”

Two reports, one from the 1940s and one from the 1960s showed that, during epidemics, staff in smallpox hospitals who had been repeatedly vaccinated sometimes developed malaise, fever, and pneumonitis without evidence of infection with smallpox or other viruses, and without evidence of allergic reaction to other agents (Howat and Arnott, 1944; Morris Evans and Foreman, 1963). In one outbreak, after investigation of other possible causes, the authors attributed the phenomena to an allergic reaction to inhaled variola. The pulmonary focus of the reaction suggests that there were significant concentrations of respirable variola in the vicinity of smallpox patients. Concentrations of respirable variola high enough to elicit allergic reactions, if true, raise a significant concern for the likelihood of airborne transmission.

## Epidemiologic evidence

Fomites, particularly exposure of laundry workers to contaminated bedding, were implicated in a few reported outbreaks (Cramb, 1951). However, during the eradication campaign careful epidemiologic investigation rarely implicated fomites as a source of infection (Fenner et al., 1988). Laundry was contaminated by scabs containing large amounts of virus, (Mitra et al., 1974) and with respiratory secretions containing virus in smaller particles (Downie et al., 1965). Very large particles with diameters greater than 50–100 μm are easily reaerosolized. Thus, the rarity of clear evidence of transmission due to fomites would be surprising, if exposure of upper respiratory mucosa to virus in large particles were an efficient means of initiating infection. However, the probability of reaerosolizing particles ≤10 μm from surfaces is extremely low because surface forces tend to bind particles more avidly the smaller the particle (Hinds, 1999). Thus, the rarity of smallpox transmission via fomites suggests that mucosal exposure was not the primary means of transmission and is consistent with a preference for infection via the lower respiratory tract.

The rarity of transmission on crowded buses and trains could be evidence that airborne transmission was not important. However, Fenner et al. (1988) state that transmission on public transport was rare because patients seldom traveled after becoming ill. They showed that transmission did occur on public transport by reporting a case of confluent smallpox who traveled early in her illness and infected five persons on a bus. If most patients who traveled were convalescent so that they no longer had virus in respiratory secretions and only shed virus in large particles from scabs, which were rarely associated with transmission of infection, (Rao et al., 1968) then lack of transmission on buses and trains was consistent with a preference for airborne transmission.

Mack (1972) emphasized that 85% of cases had clear-cut exposures to known cases. However, the remaining 15% had no obvious exposure suggesting that a small number of more distant or casual contacts transmitted infection as would be expected if smallpox were transmitted by dilute virus aerosols. For example, in the 1947 New York outbreak one secondary case was seven floors away in the hospital (Weinstein, 1947). Dispersal of smallpox downwind of hospitals was the only obvious explanation for a small number of cases in a British outbreak (Bradley, 1963; Westwood, 1963). Unexplained introductions of smallpox into Pakistani towns was greatest in towns with facilities for treatment of smallpox, (Thomas et al., 1972) which may suggest that relatively casual contact, or down wind dispersal were capable of occasionally spreading infection.

Some well-known hospital-associated outbreaks make it clear that airborne transmission at a distance of more than a few feet did occur occasionally (Wehrle et al., 1970). But, these examples were rare. However, because highly infectious disseminators are rare in other airborne infectious diseases, (Riley, 1980; Olsen et al., 2003) the rarity of superspreaders in smallpox is not an indication that transmission by less infectious cases was necessarily by a different route.

To examine whether the available data on variola aerosols is consistent with Mack's observation regarding known contacts, we can apply a standard Poisson probability model of airborne infection to estimate how long a susceptible person would need to be in a patient's room to have a reasonably high probability of contracting disease (Riley et al., 1978; Rudnick and Milton, 2003). If, we assume that inhalation and lower respiratory deposition of one PFU of variola was sufficient to cause infection, as for rabbits exposed to vaccinia and rabbit pox, (Westwood et al., 1966) and if a patient's room contained between 0.5 and 5 PFU/m^3^ in particles with a 25% lower respiratory deposition fraction (consistent with the literature discussed above), a susceptible individual breathing at 8 l/min would have needed to spend between 1.7 and 16.7 h in the patient's room to have a 63% probability of becoming infected. Outside of the patient's room, aerosol concentrations would have been much lower. If most patients stayed at home in small buildings or in hospitals without mechanically recirculated air, the risk of infection would have been significantly lower outside of patients' rooms, consistent Mack's (1972) observation that 85% of cases arose from identifiable contacts. Thus, a predominance of identifiable face-to-face contacts among cases is not strong evidence against transmission by fine particle aerosols.

The weight of evidence suggests that fine particle aerosols were the most frequent and effective mode of smallpox transmission because this would explain the relatively low mortality after variolation, the rarity of transmission by fomites, resolve the paradox of mucosal infection, and be consistent with “smallpox handler's lung” and with animal and virus aerosol experimental data. Certainly other modes of transmission occurred; full-blown disease could result from inoculation through the skin, the nasal mucosa, or the conjunctiva. Thus, smallpox cannot be classified as an “obligate” airborne infectious disease, such as tuberculosis (Riley et al., 1995) (sometimes referred to as a “true” airborne infection), because it was capable of initiating disease via infection of tissues outside of the lower respiratory tract. However, smallpox also cannot be classified as an isotropic infection (formerly termed “opportunistically” airborne infectious disease) because it appeared not to have been transmitted with equal effectiveness and virulence by all routes, whether aerosol, large droplet, or direct contact and skin inoculation. Smallpox appears to have been most effectively and virulently transmitted by fine particle aerosols and therefore should be classified as an anisotropic infection; an infection where route of transmission influences either virulence and or probability of infection, formerly called a “preferentially” airborne infectious disease.

Current recommendations for control of secondary smallpox infections emphasize transmission “by expelled droplets to close contacts (those within 6–7 feet)” (Centers for Disease Control, 2002, 2003). Recommendations include vigilant maintenance of standard, droplet, and airborne precautions. However, emphasis on spread via large droplets may reduce the vigilance with which more difficult airborne precautions are maintained. High concentrations of variola in the lung during the incubation and prodromal periods in monkeys after simulated use of variola as a bioweapon (Hahon, 1961) may indicate that first generation cases after an attack with a concentrated aerosol may be more infectious than expected based on historical data. Moreover, because airborne precautions are not routine for all hospitalized patients, and because first generation cases will probably not be initially suspected to have smallpox, it is likely that they will not be placed on airborne precautions until well into their infectious period. Therefore, the extent of transmission to a second generation in the contemporary hospital environment may be greater than expected based on historical estimates.

These considerations suggest that models of a potential smallpox attack should incorporate an aerobiological perspective to predict how the infection might propagate in the modern environment. It is particularly important to examine smallpox transmission in hospitals because hospitals have previously been identified as the major site of transmission in developed countries and ill patients will inevitably gravitate to hospitals, at least early in the outbreak before alternatives exist (Mack, 1972, 2003). Additional attention to prevention of airborne transmission in hospitals from unrecognized cases may not only be an important aspect of public health preparedness for smallpox, but may also benefit society by reduced morbidity and disruption from SARS and other emergent airborne infections.

### Conflict of interest statement

The author declares that the research was conducted in the absence of any commercial or financial relationships that could be construed as a potential conflict of interest.
